# BRG1 targeting STAT3/VEGFC signaling regulates lymphangiogenesis in colorectal cancer

**DOI:** 10.18632/oncotarget.9038

**Published:** 2016-04-27

**Authors:** Xu Zhu, Li Sun, Jingqin Lan, Linli Xu, Meng Zhang, Xuelai Luo, Jianping Gong, Guihua Wang, Xianglin Yuan, Junbo Hu, Jing Wang

**Affiliations:** ^1^ Cancer Research Institute, Tongji Hospital, Tongji Medical College, Huazhong University of Science and Technology, Wuhan, 430030, China; ^2^ Department of Oncology, Tongji Hospital, Tongji Medical College, Huazhong University of Science and Technology, Wuhan, 430030, China; ^3^ Department of Immunology, Tongji Medical College, Huazhong University of Science and Technology, Wuhan, 430030, China

**Keywords:** BRG1, lymphangiogenesis, STAT3

## Abstract

Tumor lymphangiogenesis is an important early event in tumorigenesis, one that promotes lymphatic metastasis. BRG1 (also known as SMARCA4) is a central component of the SWI/SNF chromatin-remodeling complex. In a previous work, we have reported that decreased BRG1 could promote colon cancer cell migration and invasion, and that the BRG1 expression level is negatively correlated with lymphatic metastasis. In the current study, we provide a comprehensive analysis of the role of BRG1 during lymphangiogenesis in colorectal cancer. Lymphatic vessels are more abundant in BRG1 low-expression tumors than in BRG1 high-expression tumors. We investigate the process by which BRG1 can promote VEGFC transcription and induce lymphangiogenesis *in vivo* and *in vitro*. We show that BRG1 controls lymphangiogenesis by binding to STAT3 and regulating STAT3 activation. We also prove the mechanisms through clinical samples. In summary, our demonstration of the important roles of the BRG1/STAT3/VEGFC in tumor-associated lymphangiogenesis might lead to the discovery of novel therapeutic targets in the treatment of cancers with BRG1 loss of function.

## INTRODUCTION

Lymphatic metastasis is an important factor determining the outcome of colorectal cancer (CRC) [1, 2]. When the cancer is localized, the five-year relative survival rate is 90.3%; however, this rate drops to 70.4% after the cancer undergoes lymph node metastasis [3]. Tumor lymphangiogenesis is an important early event in tumorigenesis, which can promote lymphatic metastasis [4–6]. Previous studies have reported that VEGFC/VEGFD play an important role in lymphangiogenesis [7, 8]. VEGFC or VEGFD expression level has been correlated with clinicopathologic in several studies. It has been indicated that VEGFC or VEGFD can specifically bind vascular endothelial growth factor receptor-3 (VEGFR-3), as well as promote tumor lymphangiogenesis and prevent the spread of lymphatic tumor to regional lymph nodes [4, 9]. Targeting VEGFC/VEGFR-3 pathway for lymph node metastasis and reducing the incidence of distant organ metastases have been given a therapeutic approach [9].

BRG1 (also known as SMARCA4) is a central component of the SWI/SNF chromatin-remodeling complex, which regulates transcription by remodeling the chromatin structure through the ATP-dependent disruption of DNA–histone interactions at the nucleosomes; BRG1 also features bromodomain and helicase/ATPase activity [10–12]. Mutations of BRG1 and silencing of BRG1 protein expression have been observed in several cancers; moreover, BRG1−/+ in heterozygous mice has led to increased tumor development, suggesting that BRG1 may act as a tumor suppressor [11, 12]. Previously, we have reported that decreased BRG1 expression plays a critical role in CRC metastasis; we also reported that the BRG1 loss function could promote colon cancer cell migration and invasion and that the BRG1 expression level is negatively correlated with lymphatic metastasis [13]. Given that lymphangiogenesis is an important early event and a promoting factor that induces lymphatic metastasis, we hypothesize that BRG1 may also play an important role in lymphangiogenesis. In addition, we re-analyze our previous animal study specimens and find that the lymphatic vessels are abundant in BRG1 knockdown tumors compared with BRG1 wild expression tumors. Such evidence indicates that BRG1 plays an important role in lymphangiogenesis, although the mechanism remains unknown.

In this study, we use human tumor samples as well as *in vivo* and *in vitro* studies to provide a comprehensive analysis of the role played by BRG1 during lymphangiogenesis in CRC. We compare the matched pairs of primary human colon tumors, and lymph node metastases reveal that BRG1 expression is downregulated in metastases. The lymphatic vessels are more abundant in BRG1 low-expression tumors than BRG1 high-expression tumors. For the detailed mechanism study, we used *in vivo* and *in vitro* models to investigate the process by which BRG1 promotes VEGFC transcription, then induced the lymphangiogenesis in colon cancer cell lines and xenograft tumors. We also show that BRG1 controls these phenotypes through STAT3 dependent regulation. Finally, using clinical datasets, we proved the mechanisms that are described in cell lines and xenograft tumors.

## RESULTS

### Relationship between BRG1 expression level and lymphangiogenesis in clinical samples from patients with CRC

**Figure 1:**
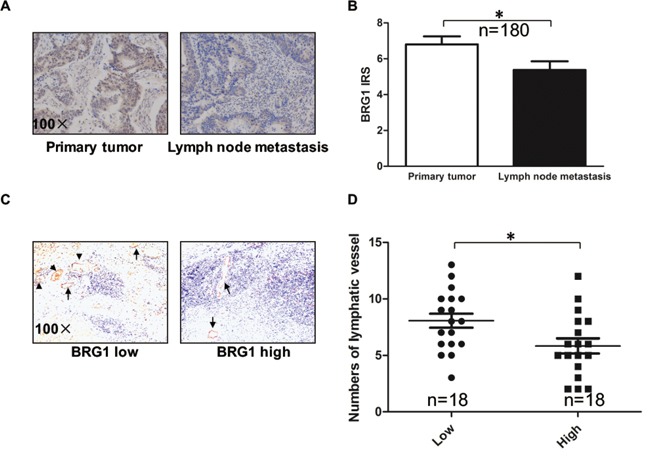
Down-regulation of BRG1 correlates with lymph node metastasis in human colorectal cancer (CRC) **A.** The BRG1 immunohistochemistry (IHC) staining of primary tumors and lymph node metastasis in CRC. Representative images of BRG1 staining were shown at 100× magnification. **B.** Quantification of BRG1 IHC staining was shown. BRG1 was over-expressed in primary tumors compared with lymph node metastasis (n=180). **C.** Representative micrographs of IHC staining of LYVE-1 (lymphatic marker) in human CRC tissues with low (n=18) and high (n=18) level of BRG1 expression. Original magnification, ×100. **D.** Quantification of lymphatic vessels IHC staining was shown. *p<0.05.

In our previous study, we reported that BRG1 expression is related with lymph node metastases [13]. To further confirm this finding, we measured the expression levels of BRG1 in lymph node metastases and primary tumors from 180 patients with lymph node metastatic CRC by immunohistonchemistry, after which we rated the amount of BRG1 staining by using an immunoreactive score (IRS) (Figure 1A and 1B). We found that BRG1 expression was lower in the lymph node metastases than in the primary tumors (*p*<0.05). Next, we analyzed the relationship between BRG1 expression level and lymphangiogenesis in CRC. We stained the lymphatic vessels by LYVE-1 in 18 BRG1 high-expression specimens and 18 BRG1 low-expression specimens (Figure 1C and 1D). We found that the BRG1 expression level was negatively related with the number of lymphatic vessels in CRC clinical samples (*p*<0.05).

### Knockdown BRG1 enhances lymphangiogenesis and CRC cell VEGFC expression

**Figure 2:**
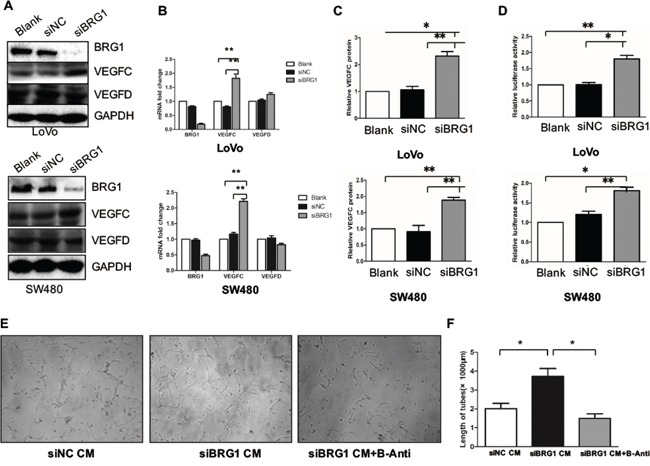
Knockdown BRG1 enhances lymphangiogenesis and CRC cell VEGFC expression **A.** Protein expression of BRG1, VEGFC, VEGFD and GAPDH after transfection with siBRG1 and siNC in LoVo and SW480 cells. **B.** Real-time PCR analysis of BRG1, VEGFC and VEGFD mRNA expression in LoVo cells and SW480 cells after transfection with siBRG1 and siNC. **C.** Quantitative expression analysis of VEGFC protein levels by ELISA in the supernatants of LoVo and SW480 cells after transfection with siNC or siBRG1. **D.** Luciferase activity assays in LoVo and SW480 cells 72 after infection of siNC or siBRG1, and the indicated pLG6-VEGFC promoter constructs. **E.** Representative images and quantifications **F.** of matrigel tube formation of human lymphatic endothelial cells (HLECs) cultured with conditioned medium derived from the indicated cells and VEGFC blocking antibody. Scale bars: 50μm. *p<0.05, **p<0.01.

In order to determine whether BRG1 may have a direct role in lymphangiogenesis, we first constructed BRG1 knockdown cells in LoVo and SW480 cell lines as well as control cells that contained empty vectors (Figure 2A). The expression levels of VEGFC and VEGFD were detected by western blot and Q-PCR. We also found that VEGFC expression level was upregulated in BRG1 knockdown cells, but VEGFD showed no change compared with the control cells (Figure 2A and 2B). Given that VEGFC could be secreted into the medium by the cancer cells, we thus checked the protein level in the medium. The ELISA analysis showed the VEGFC was increased in the medium compared with control cells medium (Figure 2C). To further confirm the regulation of BRG1 on VEGFC expression, we constructed a VEGFC promoter reporter. Relative luciferase activity assay showed that knockdown BRG1 promoted VEGFC transcriptional activity both in SW480 and LoVo cells (Figure 2D).

Previous studies have shown that VEGFC could promote tube formation in HLECs. Thus, we used BRG1 knockdown LoVo cells conditional medium (CM) to culture the HLECs, and then detected the tube formation. Meanwhile, we also used a VEGFR-3 antibody (B-Anti) to block the tube formation, in our attempt to identify the role of VEGFC in knockdown BRG1-induced lymphangiogenesis. We found that knockdown BRG1 LoVo cell CM enhanced the tube formation compared with the control CM. However, this mechanism could be blocked by B-Anti; thus, we also quantified the length of the tube (Figure 2E and 2F). Taken together, these results demonstrated that decreased BRG1 enhanced CRC cell VEGFC expression and lymphangiogenesis *in vitro*.

### Downregulating BRG1 promotes lymphangiogenesis *in vivo*

**Figure 3:**
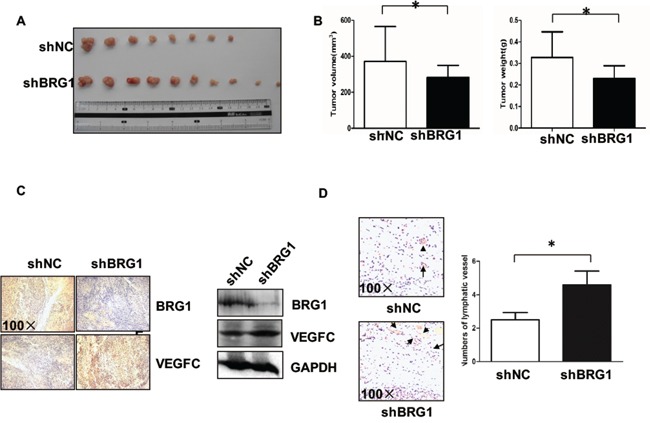
Downregulating BRG1 promotes lymphangiogenesis *in vivo* **A.** Images of the tumors from all mice in each group. Nude mice were subcutaneously injected with LoVo cells stably transfected with siNC and siBRG1. **B.** The mean tumor volumes and weight of each group were calculated. **C.** Immunohistochemical and Western blot analyses of tumor xenografts. BRG1 and VEGFC protein were examined in each group. Original magnification, ×100. **D.** (Left) Representative micrographs of tumor sections stained with anti-LYVE-1 antibody. Original magnification, ×100; (Right) Quantification of the numbers of lymphatic vessel. *p<0.05.

To confirm the effects of BRG1 on lymphangiogenesis *in vivo*, LoVo BRG1 knockdown or LoVo control cells (10^5^ cancer cells per mouse) were injected subcutaneously into the mice, which were killed after 21 days. Then, we compared the size of the tumors between the LoVo BRG1 knockdown and the LoVo control groups (Figure 3A). We quantitated the tumor volume and weight and found that they were significantly decreased when BRG1 was downregulated (Figure 3B). However, upon checking the lymphangiogenesis and VEGFC expression, IHC staining and western blot results showed that VEGFC was upregulated in the BRG1 knockdown tumors (Figure 3C); LYVE-1 staining also showed that there were more lymphatic vessels in the BRG1 knockdown tumors (Figure 3D). Taken together, these results demonstrated that decreased BRG1 played an important role in lymphangiogenesis *in vivo*.

### Relationship between BRG1 and VEGFC expression level in CRC cell lines and clinical samples

**Figure 4:**
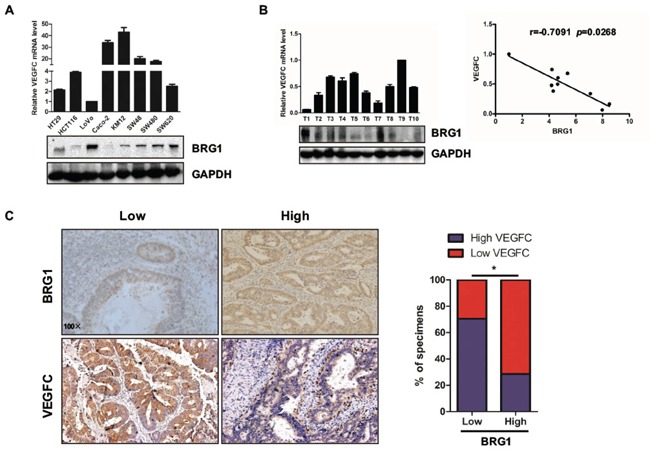
Relationship between BRG1 and VEGFC expression level in CRC cell lines and clinical samples **A.** Expression of BRG1 protein and VEGFC mRNA in 8 cultured CRC cell lines (HT29, HCT116, LoVo, Caco-2, KM12, SW48, SW480 and SW620). **B.** (Left) Analyses showing the expression of BRG1 protein and VEGFC mRNA in 10 freshly human CRC tissues. (Right) BRG1 protein expression was negatively correlated with VEGFC mRNA expression in 10 freshly human CRC tissues. **C.** (Left) BRG1 levels were negatively correlated with VEGFC expression in primary human CRC specimens (*n*=31). Micrographs of BRG1 and VEGFC expression level in human CRC tissues. Original magnification, ×100. (Right) Percentage of CRC specimens with low or high BRG1 expression relative to VEGFC expression. *p<0.05.

To further evaluate the relationship between BRG1 and VEGFC expression in CRC, we measured the expression levels of BRG1 by western blot in eight different CRC cell lines (HT29, HCT116, LoVo, Caco-2, KM12, SW48, SW480, and SW620). Data showed that BRG1 expression level was negatively related with VEGFC transcriptional expression level (Figure 4A). Next, we analyzed the relationship between BRG1 and VEGFC expression in the samples from CRC patients, and found that BRG1 expression level was negatively related with VEGFC transcriptional expression level in 10 samples (Figure 4B). Furthermore, the IHC staining of LYVE-1 in 31 samples also indicated that BRG1 expression level was negatively related with VEGFC expression level (Figure 4C).

### BRG1 regulation of STAT3/VEGFC signaling mediates lymphangiogenesis

STAT3 signaling plays an important role in cancer lymphangiogenesis and metastasis [15, 16]. Previous studies have reported that p-STAT3 inhibitor S3I-201 treatment inhibited lymph node metastasis and improved metastatic breast cancer outcomes. Two primary transcription factors that directly regulate VEGFC expression are STAT3 and HIF-1a [17]. Such evidences prompted us to investigate whether BRG1 modulated STAT3/VEGFC signaling to influence lymphangiogenesis in CRC.

**Figure 5:**
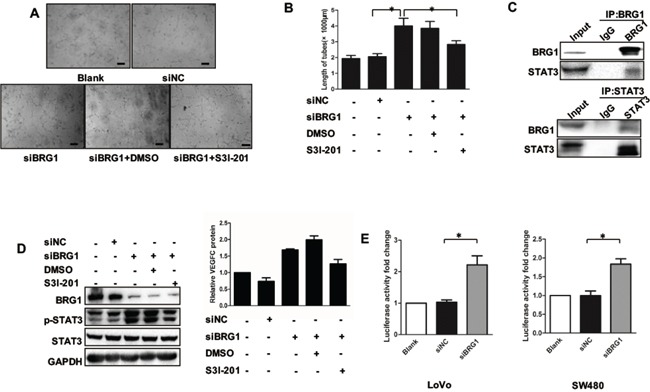
BRG1 regulation of STAT3/VEGFC signaling mediates lymphangiogenesis **A.** Representative images and quantifications **B.** of tube formation of human lymphatic endothelial cells (HLECs) cultured with conditioned medium derived from LoVo cells. DMSO was used as vehiche control. Scale bars: 50μm. **C.** Immunoprecipitation using anti-BRG1 or anti-STAT3 antibodies was performed in LoVo cells, followed by immunoblotting with the indicated antibodies. **D.** (Left) Western blot analysis of BRG1, STAT3, p-STAT3 expression after transfection with siNC and siBRG1 in the presence or absence of S3I-201 (STAT3-specific inhibitor) in SW480 cells. (Right) Quantitative expression analysis of VEGFC protein levels by ELISA in the supernatants of SW480 cells after transfection with siNC and siBRG1 in the presence or absence of S3I-201 (STAT3-specific inhibitor) in SW480 cells. *p<0.05, **p<0.01. **E.** The luciferase activity fold change of STAT3 pathway reporter in LoVo and SW480 cells.

First, we employed a rescue experiment by co-transfecting SW480 cells with siBRG1 and STAT3 inhibitor S3I-201, after which we harvested the CM and added the CM into cultured HLECs. These procedures were done to further confirm whether BRG1 reduction increases STAT3-mediated effects on lymphangiogenesis and VEGFC expression. Indeed, we found that transfection of siBRG1 SW480 CM promoted lymphangiogenesis in HLECs. However, these effects were abrogated when SW480 cells were treated with S31-201 (Figure 5A, 5B). Such findings suggested that BRG1 reduction promoted lymphangiogenesis depending on the STAT3 signaling. In identifying the mechanism of the BRG1 regulation of STAT3 signaling, we referred to co-immunoprecipitation (Co-IP) and found that BRG1 and STAT3 were involved in the same complex (Figure 5C). We then conducted another rescue experiment by transfecting SW480 cells with siBRG1 and the STAT3 inhibitor, S3I-201. We observed that the reduction of BRG1 expression promoted STAT3 activation and VEGFC expression. However, these effects were abrogated when cells were co-treated with S3I-201 (Figure 5D). We checked the STAT3 pathway activation by checking the STAT3 pathway reporter in BRG1 knockdown and normal condition, the data showed knockdown of BRG1 increased the STAT3 pathway activation both in LoVo and SW480 cells (Figure 5E). Taken together, these results strongly suggested that BRG1 modulated CRC cell-induced lymphangiogenesis through its effects on STAT3/VEGFC signaling in CRC cells.

## DISCUSSION

Several studies have revealed that tumor lymphangiogenesis is correlated with lymph node metastasis in experimental cancer models and human cancers [9]. Metastatic tumor cells can continue promoting lymphatic vessel growth even after their metastasis to sentinel lymph nodes, thereby promoting the further spread of cancer [18]. Cancer cell can release the VEGFC and VEGF-D, which are the specific lymphangiogenesis factors identified. Moreover, a large number of clinical studies have shown the correlation between tumor expression of VEGFC or VEGF-D and lymph node metastasis [6].

BRG1, a central component of the SWI/SNF chromatin-remodeling complex, is involved in either transcriptional activation or transcriptional repression of a subset of genes [19]. In addition, BRG1 has been shown to interact with the tumor suppressor, p53, thereby leading to the transcriptional activation of target genes, including tumor suppressors such as prohibitin, TopBP1 and HIC1 [20–22]. In our previous study, we have shown that mutant or loss functional BRG1 promoted colon cancer metastasis *in vitro* and *in vivo*[13]. In this study, we investigated the function of BRG1 in cancer-related lymphangiogenesis. We found that downregulation of BRG1 promoted both cancer cell VEGFC production and cancer cell-induced lymphangiogenesis in colon cancer. *in vivo* and *in vitro* data proved our assumption that BRG1 plays a critical role in cancer lymphangiogenesis.

STAT3 activation has been linked to VEGFC expression and oncogenesis, but the mechanisms underlying such hyperactivity are not well understood. In this study, we found that BRG1 can bind to STAT3 and downregulated BRG1-induced STAT3 signaling activation. Our previous study and some other reports have shown that BRG1 loss function mutation is a common mechanism in several kinds of cancers, and this may be one of the reasons behind STAT3 hyperactivity in cancers. In this study, we demonstrated the importance of BRG1 in lymphangiogenesis through its regulation of STAT3/VEGFC signaling. We also investigated how the BRG1's regulation of the tumor-induced lymphangiogenesis depended on STAT3 and VEGFC, which is a mechanism that could be blocked by STAT3 inhibitor or the VEGFC antibody.

Tumor-associated lymphangiogenesis has now been firmly established as a novel mechanism for cancer progression, and the blockade of tumor-induced lymphatic vessel growth within metastatic lymph nodes might prevent further cancer spread to distant organs [4, 18]. Many preclinical datasets have indicated that the blockade of the VEGFC/VEGFR-3 pathway inhibits tumor spread to lymph nodes and beyond [1]. BRG1 loss function mutation has been found in several kinds of cancers, and our demonstration of the important role of the BRG1/STAT3/VEGFC in tumor-associated lymphangiogenesis might lead to the discovery of novel therapeutic targets to treat cancer with BRG1 loss of function. However, in this study, the mechanism by which BRG1 regulates STAT3 activation remains unknown. Hence, this topic should be further explored in future studies.

## MATERIALS AND METHODS

### Cell lines and cell culture

All CRC cell lines (LoVo, SW480, HT29, HCT116, Caco-2, KM12, SW48 and SW620) and the HEK293T cell line were obtained from American Type Culture Collection (ATCC, Manassas, VA, USA). The cells were cultured at 37°C with 5% CO2 in Dulbecco's modified Eagle's medium (DMEM) supplemented with 10% fetal bovine serum (HyClone, Logan, Utah, USA) and penicillin/streptomycin. Human lymphatic endothelial cells (HLECs) were purchased from PriCells Biomedical Technology Co., Ltd. (Wuhan, China), and cultured according to the instructions of the manufacturer. In all related experiments, the fourth passage of HLECs was used.

### RNA interference

The siRNA targeting human BRG1 (siBRG1), and siRNA-negative control (siNC) were synthesized and purified by RiboBio (Ribobio Co. Ltd, Guangzhou, China). siRNA was transfected by using Lipofectamine 2000 (Invtirogen, Carlsbad, CA, USA) and medium was replaced 6h after transfection. A final concentration of 100nM siBRG1 and siNC was used, and the expression and mRNA were checked at 48h after transfection.

### Luciferase activity assay

A DNA fragment containing VEGFC promoter (from −688bp to +201bp) was amplified from human genomic DNA with primers 5′-GGGGTACCCACAGACCAAGGGAGAGAGG-3′ and 5′-CCAAGCTTCTCACAGGAAACCGGACATC-3′, and then cloned into luciferase report vector pGL6. Luciferase activity assay was performed as previously described using the Dual-Luciferase Reporter Assay System (Promega, Madison, WI, USA) according to the instructions of the manufacturer. LoVo and SW480 cells were transient transfected with siNC, siBRG1, pLG6-VEGFC promoter, and Renilla luciferase vector. Cell lysate was collected for luciferase activity assay 48 h after transfection. Firefly luciferase activity was normalized to the corresponding Renilla luciferase activity. All experiments were performed in triplicate.

### Immunoprecipitation and Western blot

Immunoprecipitation and Western blot were performed as described previously [14], using anti-p-STAT3 antibody (Cell Signaling Technology, Danvers, Massachusetts, USA), anti-VEGFC antibody (Abgent, San Diego, CA, USA), as well as anti-BRG1, anti-STAT3, anti-GAPDH antibodies (Santa Cruz Biotechnology, Dallas, TX, USA). GAPDH was used as a loading control. STAT3 inhibitors S3I-201 came from Selleck (Shanghai, China).

### Tissue specimens, immunohistochemistry and IHC images analysis

The CRC samples used in this study were histopathologically and clinically diagnosed at Tongji Hospital, Wuhan, China. Procedures involving human subjects were approved by the Huazhong University of Science and Technology Ethics Committee, and a formal form was explained to each subject to ensure their full understanding and consent. The clinicopathological characteristics of the samples and IHC image analysis were shown. The level of BRG1 staining was evaluated by IRS, which was calculated by multiplying the scores of staining intensity and the percentage of positive cells. Based on the IRS, BRG1 staining patterns were defined as low (IRS: 0–4) and high (IRS: 6–12). Lymphatic vessel density (LVD) was determined according to the methods described by Gombos et al. and Zhang et al. using anti-LYVE-1 antibody (Millipore, Bedford, Massachusetts, USA). The sections were reviewed and scored separately by two independent pathologists.

### Human lymphatic endothelial cell tube formation assay

HLECs tube formation assay was performed by pipetting 30 μL/well matrigel (BD Biosciences, Bedford, Massachusetts, USA) and 20 μL/well serum-free ECM into each well of a 96-well plate; this was then polymerized for 30 min at 37°C. Next, HLECs (7×10^3^) in 100 μL of conditioned medium were added to each well and incubated at 37°C with 5% CO2 for 6 h. The tube morphogenesis was assessed by phase-contrast microscopy. The capillary tubes were quantified by measuring the total length of the tube structures in three random fields. The average length of tube field was calculated, and the conditioned medium was collected at 48 h after transfection. VEGFC blocking antibodies were added to the conditioned medium after the conditioned medium was added to the wells.

### RNA isolation and real-time PCR

Total mRNA was isolated using the TRIzol reagent (Invitrogen). Complementary DNA synthesis was achieved using a kit (One-Step PCR, Qiagen, Hilden, Germany) and then subjected to real-time PCR according to the protocols of the manufacturer. The primers used included the following: (forward: 5′-AATGCCAAGCAAGATGTCGAT-3′; reverse: 5′- GTTTGAGGACACCATTGACCATA-3′), VEGFC (forward: 5′- GGCTGGCAACATAACAGAGAA-3′; reverse: 5′- CCCCACATCTATACACACCTCC-3′), VEGFD (forward: 5′- ACTCAGTGCAGCCCTAGAGAA-3′; reverse: 5′- GAACACGTTCACACAAGGGG-3′), STAT3 (forward: 5′- CAGCAGCTTGACACACGGTA-3′; reverse: 5′- AAACACCAAAGTGGCATGTGA-3′), GAPDH (forward: 5′- GGAGCGAGATCCCTCCAAAAT-3′; reverse: 5′- GGCTGTTGTCATACTTCTCATGG-3′). A triplicate sample was analyzed in each group.

### Preparation of conditioned medium (CM) and ELISA analysis

The LoVo and SW480 cells were seeded in 6-well plates and then treated with siNC and siBRG1 after 12 h. After 48 h, the cells were washed three times in PBS and incubated in serum-free medium for 6 h. The conditioned medium was collected and stored at −80°C. VEGFC in the culture supernatants of tumor cells were quantified using the ELISA kit. These procedures were done to facilitate the analysis of human VEGFC according to the instructions of the manufacturer (R&D Systems).

### Xenografted tumor model

Male BALB/c nude mice (4-5 weeks of age, 18-20g) were purchased from Beijing HFK Laboratory (Beijing, China). All experimental procedures were conducted by following the Animal Study Guidelines of Huazhong University of Science and Technology. The BALB/c nude mice were randomly divided into two groups. The siNC, and siBRG1 (shBRG1)-transduced LoVo cells (5×10^5^) were injected subcutaneously into the flank of the mice. The mice were sacrificed after 3 weeks, and tumors were collected and tumor weight was measured. The tumor volume was calculated by formula: volume=length×width^2^/2.

### Statistical analysis

All statistical analyses were carried out using SAS13.0 statistical software. Data were presented as means±SEM. Data were calculated and two-tailed Student's t-test was performed. Correlation analyses were done using Spearman's rank test. Values of *p*<0.05 were considered statistically significant.
